# Comparing SARS-CoV-2 Testing in Anterior Nasal Vestibular Swabs vs. Oropharyngeal Swabs

**DOI:** 10.3389/fcimb.2021.653794

**Published:** 2021-07-07

**Authors:** Meiyan Li, Ruoyan Wei, Yaling Yang, Taiwen He, Yinzhong Shen, Tangkai Qi, Tian Han, Zhigang Song, Zhaoqin Zhu, Xiaopeng Ma, Yixiao Lin, Yasheng Yuan, Keqing Zhao, Hongzhou Lu, Xingtao Zhou

**Affiliations:** ^1^ Department of Ophthalmology and Optometry, Eye and ENT Hospital, Fudan University, Shanghai, China; ^2^ NHC Key Laboratory of Myopia, Fudan University, Shanghai, China; ^3^ Shanghai Research Center of Ophthalmology and Optometry, Shanghai, China; ^4^ Department of Ophthalmology, Shanghai Public Health Clinical Center, Shanghai, China; ^5^ Department of Infectious Diseases and Immunology, Shanghai Public Health Clinical Center, Shanghai, China; ^6^ Department of Pathogen Diagnosis and Biosafety, Shanghai Public Health Clinical Center, Shanghai, China; ^7^ Department of Laboratory Medicine, Shanghai Public Health Clinical Center, Shanghai, China; ^8^ Shanghai Research Institute of Acupuncture and Meridian, Shanghai University of Traditional Chinese Medicine, Shanghai, China; ^9^ Department of Otolaryngology, Eye and ENT Hospital, Fudan University, Shanghai, China

**Keywords:** SARS-CoV-2, COVID-19, nasal vestibule, diagnostics, transmission

## Abstract

**Purpose:**

To investigate the sensitivity of SARS-CoV-2 testing in specimens collected from the anterior nasal vestibules of COVID-19 patients.

**Methods:**

A cross-sectional analysis was performed on 30 patients with a confirmed diagnosis of COVID-19 at the Shanghai Public Health Clinical Center from March 14, 2020 to March 21, 2020. Paired specimens were collected from both the anterior nasal vestibule and the oropharynx from all patients. All specimens were tested for SARS-CoV-2 using reverse transcription-polymerase chain reaction (RT-PCR) assays.

**Results:**

Of the 30 patients with confirmed COVID-19, 17 patients (56.7%) tested positive for SARS-CoV-2 when oropharyngeal specimens were used, while 20 patients (66.7%) tested positive when nasal swab specimens were used. There was no statistically significant difference in sensitivity between the two methods.

**Conclusions:**

Respiratory swabs collected from the nasal vestibule offer a less invasive alternative to oropharyngeal swabs for specimen collection in the detection of SARS-CoV-2 infection, and have adequate sensitivity.

## Introduction

An outbreak of a novel coronavirus, SARS-CoV-2, began in December 2019 and has since spread around the world as a global pandemic with substantial morbidity and mortality. It has posed significant threats to international health.

Rapid and accurate detection of SARS-CoV-2 is critical in controlling the outbreak and preventing the spread of the disease. As recommended by World Health Organization (WHO), specimens should be collected from the naso-or-oropharynx using swabs or washes to diagnose and monitor COVID-19 patients (World Health Organization, 2020). However, such collection requires close contact with patients, which increases the risk of transmission of the virus from the patient to the healthcare worker. Additionally, this method of collection causes discomfort, pain, and sometimes even bleeding, hence, routine specimen collection from the nasopharynx or oropharynx may not be desirable for monitoring COVID-19.

Anterior nasal specimens may offer a convenient alternative. It has been reported that viral RNA was detected in nasal swabs collected from the middle concha (Zou et al., 2020) or anterior to the inferior concha (Péré et al., 2020). Péré et al. described a molecular detection of SARS-CoV-2 by inserting the swab in the nostril until it hit the inferior concha, and reported a sensitivity of 89.2% and a specificity of 100.0% (Péré et al., 2020). Gertler et al. reported a self-collected mixed sample of oral/nasal/saliva are reliable alternatives to professional collected pharyngeal samples (Gertler et al., 2021). However, the viral load purely from the more anterior nasal cavity is reported rarely. The benefits of collection from this site are that it is less invasive and can be self-collected by patients.

We conducted a cross-sectional study to evaluate the positive rate of SARS-CoV-2 testing in the nasal vestibule, the most anterior part of the nasal cavity, from patients with confirmed COVID-19. In this study, we aimed to report the presence of SARS-CoV-2 in the nasal vestibule and the sensitivity of testing this particular specimen for diagnosis of SARS-CoV-2 infection.

## Material and Methods

### Study Design and Patients

In this cross-sectional study, we recruited 30 patients with confirmed COVID-19 diagnoses who were hospitalized at the Shanghai Public Health Clinical Center (SPHCC), China between March 14, 2020 and March 21, 2020. Diagnoses were confirmed according to the Chinese national guidelines for COVID-19. Positive tests were confirmed by the Chinese Center for Disease Control and Prevention (China CDC). All patient cases were classified as mild to critically ill according to COVID-19 Guidelines (5^th^ version) from the National Health Commission of China. Specifically, the mild cases were defined as having slight clinical symptoms with normal radiologic findings in both lungs; the typical cases were defined as presenting with fever and/or respiratory symptoms plus pneumonia on radiography.

All patients provided signed informed consent in advance for the collection of nasal swab specimens. This study adhered to the Declaration of Helsinki and was approved by the Ethics Committee of SPHCC (yj-2020-s047-01) and Eye and ENT Hospital of Fudan University (No.2020020).

### Main Outcome Measures

The demographics, epidemiological history, clinical characteristics, symptom onset, laboratory findings, chest computed tomographic (CT) scans, serum antibodies, and treatments were obtained from patients’ electrical medical records.

**Figure 1:**
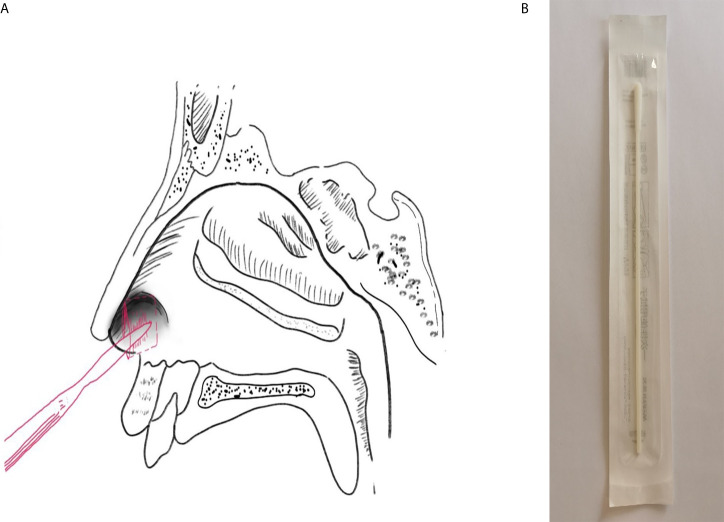
Sampling. **(A)** Specimen collection location of the nasal vestibule swab; **(B)** Swab used in the present study.

The paired specimens (swabs from the anterior nasal vestibule and oropharynx) were collected in every patient. Specifically, the nasal swab was inserted to 1 cm inside the nostril and the specimen was sampled by firmly rotating the swab and leaving it in place for 10 to 15 seconds (**Figure 1**). Specimens from each nasal vestibule were taken by the same doctor at SPHCC. The oropharyngeal swabs were collected in accordance with the China CDC guidelines. The samples were collected at a median of 1 day after hospitalization (range: 1–37 days). For all subjects, the nucleic acid tests for oropharyngeal swabs were positive at least once after this sampling.

We extracted RNA from the swabs and conducted real-time reverse transcription-polymerase chain reaction (RT-PCR) for SARS-CoV-2 as previously described (Huang et al., 2020) by using reagents provided by Da An Gene Co., Ltd. (Sun Yat-Sen University, http://en.daangene.com).

### Statistical Analysis

Data were analyzed using SPSS version 22.0 for Windows (SPSS Inc, Chicago, IL, USA). Continuous variables are presented as mean ± standard deviation (SD), and categorical variables, as frequencies. Sensitivity is defined as: (number of true positives)/(number of true positives + number of false negatives). The paired chi-square test was adopted to compare the sensitivity between groups. A two-tailed *p*-value of <0.05 was considered statistically significant.

## Results

A total of 30 Chinese patients diagnosed with COVID-19 were enrolled, of whom, 26 were imported cases from the United Kingdom (7), Italy (4), Spain (4), America (4), Iran (3), France (3), and Swiss (1). The median age was 27 years (range: 16-75). There were 18 males and 12 females. The median number of days since self-reported symptom onset was 7 days (range: 1-41). Nine (30.0%) cases were classified as typical type, while 21 (70.0%) were classified as mild type at the time of sampling.

**Table 1** and **Supplementary Table 1** show the patients positive for SARS-CoV-2 infection using the oropharyngeal and nasal swab, respectively. Sensitivity for the detection of SARS-CoV-2 in infected patients was 56.67% (95% confidence interval [CI]: 37.66%-74.02%) for oropharyngeal swabs and 66.67% (95%CI: 47.14%-82.06%) for nasal swabs. No significant difference in sensitivity was found (*p* = 0.508). The agreement kappa coefficient was 0.372.

**Table 1:** Detection of SARS-CoV-2 infection using nasal and oropharyngeal swabs in patients with confirmed COVID-19.

Oropharyngeal	+	-
Nasal		
+	14	6
–	3	7

Among 20 patients with SARS-CoV-2-positive nasal vestibule specimens, 6 patients (30.0%) had no respiratory symptoms such as nasal congestion, rhinorrhea, sneezing, and coughing. The only symptom reported by patients #16, #29, and #30 was fever. Patient #4 had a headache for three days. Patient #22 had a fever 6 days before admission which relieved without medication; at admission, he only had a headache. Patient #28 had fatigue without any other symptoms for 1 day.

## Discussion

Nasal vestibule swab sampling is a promising alternative for the detection of SARS-CoV-2 infection, as this collection site is more accessible and more comfortable for the patient compared to the oropharynx. In this study, the sensitivities of SARS-CoV-2 molecular testing in swabs collected from the nasal vestibule vs. the oropharynx were compared, and nasal vestibule swabs have been shown to have equivalent sensitivity to oropharyngeal swabs.

In this study, SARS-CoV-2 was detected in the nasal vestibular swab specimens in 66.67% of patients and there was no significant difference in sensitivity between the nasal vestibule and oropharyngeal specimen. Wang et al. (2020) and Péré et al. (2020) reported a high sensitivity of SARS-CoV-2 detection in deep nasal swabs as compared to pharyngeal sampling, which corresponds to the findings of the current study. Previous studies have also indicated that the nasal swab can be used for the detection of other respiratory virus infections, such as a respiratory syncytial virus (Lambert et al., 2008; Meerhoff et al., 2010). Besides equivalent sensitivity, there are other advantages in using nasal vestibule specimens. First, the specimens can be easily and safely self-collected by patients, thereby minimizing the risk of transmission to a healthcare worker during collection. Additionally, the ease of self-collection makes screening in the face of limited staffing possible. Furthermore, nasal vestibule sampling is well tolerated and is appropriate especially for frail individuals. Therefore, the nasal vestibule specimens are suitable for the diagnosis and monitoring of COVID-19.

In six patients in this study, SARS-CoV-2 RNA was detected in the nasal vestibule but not detected in the oropharynx, and the concordance between the two methods was relatively poor. One possible reason is individual variation in the distribution of SARS-CoV-2. The viral load could be higher in the nasal vestibule than the oropharynx for these patients. The density and persistence of the SARS-CoV-2 virus vary with location in the airways (Hernes et al., 2013) and depends on receptor accessibility. Research has shown that the SARS-CoV-2 entry receptor, ACE2, and viral entry-associated protease, TMPRSS2, were highly co-expressed in the nasal goblet and ciliated cells (Sungnak et al., 2020). ACE2 was also expressed in olfactory support cells, stem cells, and perivascular cells (Brann et al., 2020). Therefore, the nasal cavity is susceptible to SARS-CoV-2 infection and can be niches for viral replication. Adding a nasal vestibule sample to an oropharynx sample may increase the diagnostic yield of SARS-CoV-2.

In this study, SARS-CoV-2 RNA was also detected in nasal vestibule specimens of six patients without upper respiratory tract symptoms, suggesting the potential for SARS-CoV-2 transmission from asymptomatic or minimally symptomatic patients. This finding is consistent with that of Zou et al. (2) who found the viral load in deep nasal swabs in one asymptomatic patient was similar to that in the symptomatic patients. Respiratory viruses in the nasal cavity are believed to be spread through respiratory droplets when patients cough or sneeze. Our study proved that respiratory droplets containing SARS-CoV-2 could be detected in the nasal vestibule even during normal breathing, and therefore, may be transmitted directly or indirectly even among patients without respiratory symptoms. This further reinforces the use of masks as a control measure. The finding also sheds light on the fact that direct contact with patients’ noses or nasal secretions during diagnostic and therapeutic procedures poses a serious risk to health professionals, especially otolaryngologists. It is advisable to avoid all non-emergent upper airway surgeries and non-essential examinations.

This study has some limitations. First, the sample size was relatively small. However, because the COVID-19 is highly contagious and incredibly lethal, the subjects had to be enrolled in limited time to protect the practitioners and to obey the policy of SPHCC. Almost all available COVID-19 patients in the ward were collected. Secondly, since all enrolled patients had a confirmed diagnosis of COVID-19, the specificity cannot be evaluated in this study. Further evaluation of this new detection method in a larger population is needed. Additionally, because this is a cross-sectional study, we were unable to determine the change over time of virus detectability in the two specimens.

In conclusion, our results have demonstrated that swabbed specimens from the nasal vestibule have a relatively high sensitivity for detecting SARS-CoV-2 infection. This collection method is also less invasive and more readily allows for patient self-collection. As there are no sacrifices in testing sensitivity and has added advantages, the anterior nasal vestibule is a promising alternative site for specimen collection in the monitoring of COVID-19.

## Data Availability Statement

The original contributions presented in the study are included in the article/**Supplementary Material**. Further inquiries can be directed to the corresponding authors.

## Ethics Statement

The studies involving human participants were reviewed and approved by Ethics Committee of Shanghai Public Health Clinical Center (yj-2020-s047-01) and Eye and ENT Hospital of Fudan University (No.2020020). The patients/participants provided their written informed consent to participate in this study.

## Author Contributions

Research concept and design: XZ and HL. Data collection and performance of the research: all authors. Analysis and interpretation of data: ML, RW, YLY, and TH. Writing of the manuscript: ML and RW. Critical revision of the manuscript: XZ, HL, and KZ. Supervision: XZ. All authors contributed to the article and approved the submitted version.

## Funding

This study was supported by the Second Batch of Emergency Project of the Shanghai Science and Technology Committee (20411950200) and the Special Emergency Project for the Prevention and Treatment of COVID-19 with Traditional Chinese Medicine in Shanghai (2020NCP001).

## Conflict of Interest

The authors declare that the research was conducted in the absence of any commercial or financial relationships that could be construed as a potential conflict of interest.
