# Supplementary material for: Comparing SARS-CoV-2 Testing in Anterior Nasal Vestibular Swabs vs. Oropharyngeal Swabs
Source: Front Cell Infect Microbiol. 2021 Jul 7;11:653794. doi: 10.3389/fcimb.2021.653794 (PMC8293915; doi:10.3389/fcimb.2021.653794)
Supplement: Supplementary file 1 [file Table_1.docx]

Supplementary Table 1. The results of oropharygeal and nasal vestibular specimens of each patient.

| Patients ID | Oropharygx | Nasal vestibule | Patients ID | Oropharygx | Nasal vestibule |
| --- | --- | --- | --- | --- | --- |
| 1 | - | - | 16 | + | + |
| 2 | - | - | 17 | - | - |
| 3 | - | - | 18 | + | + |
| 4 | + | + | 19 | - | + |
| 5 | - | - | 20 | - | - |
| 6 | - | - | 21 | + | + |
| 7 | + | - | 22 | - | + |
| 8 | + | + | 23 | - | + |
| 9 | + | + | 24 | + | + |
| 10 | + | - | 25 | + | + |
| 11 | - | + | 26 | + | + |
| 12 | + | + | 27 | + | + |
| 13 | + | + | 28 | - | + |
| 14 | + | - | 29 | + | + |
| 15 | - | + | 30 | + | + |
